# Construction of a Dual-Fluorescence Reporter System to Monitor the Dynamic Progression of Pluripotent Cell Differentiation

**DOI:** 10.1155/2016/1390284

**Published:** 2016-11-24

**Authors:** Wu-Sheng Sun, Ju-Lan Chun, Jeong-Tae Do, Dong-Hwan Kim, Jin-Seop Ahn, Min-Kyu Kim, In-Sul Hwang, Dae-Jin Kwon, Seong-Soo Hwang, Jeong-Woong Lee

**Affiliations:** ^1^Biotherapeutics Translational Research Center, Korea Research Institute of Bioscience and Biotechnology, Daejeon 305-806, Republic of Korea; ^2^Department of Animal Science and Biotechnology, College of Agriculture and Life Science, Chungnam National University, Daejeon 305-764, Republic of Korea; ^3^Department of Stem Cell and Regenerative Biology, College of Animal Bioscience and Technology, Konkuk University, Seoul, Republic of Korea; ^4^Animal Biotechnology Division, National Institute of Animal Science, Wanju 565-851, Republic of Korea

## Abstract

Oct4 is a crucial germ line-specific transcription factor expressed in different pluripotent cells and downregulated in the process of differentiation. There are two conserved enhancers, called the distal enhancer (DE) and proximal enhancer (PE), in the 5′ upstream regulatory sequences (URSs) of the mouse* Oct4* gene, which were demonstrated to control* Oct4* expression independently in embryonic stem cells (ESCs) and epiblast stem cells (EpiSCs). We analyzed the URSs of the pig* Oct4* and identified two similar enhancers that were highly consistent with the mouse DE and PE. A dual-fluorescence reporter was later constructed by combining a DE-free-*Oct4*-promoter-driven EGFP reporter cassette with a PE-free-*Oct4*-promoter-driven mCherry reporter cassette. Then, it was tested in a mouse ESC-like cell line (F9) and a mouse EpiSC-like cell line (P19) before it is formally used for pig. As a result, a higher red fluorescence was observed in F9 cells, while green fluorescence was primarily detected in P19 cells. This fluorescence expression pattern in the two cell lines was consistent with that in the early naïve pluripotent state and late primed pluripotent state during differentiation of mouse ESCs. Hence, this reporter system will be a convenient tool for screening out ESC-like naïve pluripotent stem cells from other metastable state cells in a heterogenous population.

## 1. Introduction

The population in a culture of pluripotent cells is not homogenous; therefore, an appropriate reporter system, which can screen out the pluripotent stem cell (PSCs) from other metastable stem cells or even completely differentiated somatic cells, is necessary. To date, many reporters have been constructed by combining the promoter from candidate pluripotent genes, such as* Nanog* [1],* Rex-1* [2], or* Oct4* [3] and a fluorescent protein. Next, by monitoring the fluorescence signal, the expression of pluripotency-related genes could be determined and the pluripotent cells could be easily isolated from the heterogenous cell population without additional staining processes [4].

*Oct4* (also known as* Oct3* or* POU5F1*) is one of the well-known reporter genes because its expression is restricted in pluripotent cells and germ cells [5]. Upon differentiation of PSCs,* Oct4* expression was gradually reduced and finally silenced along with epigenetic modifications [6]. The silenced* Oct4* in differentiated somatic cells can be reactivated by several reprogramming processes such as fusion-induced reprogramming, somatic cell nuclear transfer (SCNT), or generation of induced pluripotent stem cells (iPSCs) [7, 8], suggesting the importance of* Oct4* in maintenance and self-renewal of pluripotent cells. An* Oct4* reporter system, constructed by integrating the* Oct4* promoter into GFP, can be used as an efficient marker to mimic the endogenous* Oct4* gene expression in mouse [9]. So far, a variety of* Oct4*-promoter-driven* GFP* or* EGFP* reporters have been used in mouse [10, 11], human [12, 13], cattle [14, 15], rabbit [16, 17], zebrafish [18], medaka [19], and pig [20, 21] models.

PSCs have been classified into at least two states: naïve and primed pluripotent states [22, 23]. Mouse embryonic stem cells (mESCs) are referred to as an earlier or naïve pluripotent state, while mouse epiblast stem cells (EpiSCs) correspond to a later or “primed” pluripotent state. All of the cells of the two types of pluripotent stem cells express pluripotency genes, such as* Oct4* and* Nanog*, and can differentiate into cells of all three germ layers* in vitro*, but they are also distinct in many aspects. Naïve PSCs are characterized by formation of compact and dome-like colonies [24], are dependent on leukemia inhibitory factor (LIF) [25], contain two active X chromosomes (XaXa) [26, 27], and, most notably, efficiently contribute to chimeras [28]. In contrast, primed PSCs form flat colonies, respond to basic fibroblast growth factor (bFGF) and activin instead of LIF, and have an inactivated X chromosome (XaXi) [28, 29].

To distinguish between naïve and primed PSCs, transcriptome analysis [30] or immunostaining has typically been conducted. However, little is known about porcine-specific pluripotency markers, which is a basic obstacle to studying PSCs in pigs using this approach [4].* Oct4*-GFP reporter systems can be a good marker for PSC studies [31], but* Oct4*-GFP itself seems infeasible for categorizing the two PSC types because* Oct4* is expressed in both naïve and primed PSCs [32]. Interestingly, previous reports indicated that the expression of mouse* Oct4* in the two different PSC states is regulated by two independent enhancers. In naïve PSCs,* Oct4* was primarily controlled by the distal enhancer (DE), whereas, in primed PSCs, it is driven by its proximal enhancer (PE) [33, 34]. Based on these studies, we established a dual reporter system using the DE or PE deleted upstream regulatory sequences (URSs) of pig* Oct4* to drive EGFP and mCherry (RFP) gene expression. Before this reporter is directly used in pig, firstly, we tested it in three types of defined mouse PSCs with different levels of pluripotency. We expect that this reporter system can be a useful tool for screening out naïve PSCs from primed PSCs and for monitoring the dynamic progression of cell differentiation.

## 2. Materials and Methods

The use of animals in this study was approved by the Institutional Animal Care Committee of the Korea Research Institute of Bioscience and Biotechnology and the current guidelines on animal care were followed. All chemicals used in this study were purchased from Sigma Aldrich (USA), unless otherwise stated.

### 2.1. Alignment of* Oct4* URSs in Cow, Human, Mouse, and Pig

The sequences of the* Oct4* URS for cow (chr23: 27,766,782–27,769,892), human (chr6: 31,170,621–31,173,790), mouse (chr17: 35,503,313–35,506,099), and pig (chr7: 27,259,932–27,262,689) were obtained from UCSC (https://genome.ucsc.edu/). The sequences in the gap region in the cow* Oct4* URS (chr23: 27,766,985–27,767,084) was obtained from previous study [36]. Comparison of each sequence was performed with DNAMAN (Lynnon Biosoft, USA). The conserved region was found with the mVISTA program in LAGAN mode with default parameters [37]. Additional 1,000 bp sequences downstream of the translation initiation site of the* Oct4* gene were selected together with their URS mentioned above and, when analyzed, the distribution of the CpG islands was used as a reference [38].

### 2.2. Construction of Porcine Oct4-EGFP/mCherry Reporter Vectors

Pig umbilical cord was collected from the National Institute of Animal Science (Suwon, Korea). The collected tissue was taken to the laboratory and immediately washed twice with Dulbecco's phosphate-buffered saline (DPBS) (Welgene, Korea) and frozen in liquid nitrogen until used for DNA isolation. A 5.6 kbp regulatory region of the porcine* Oct4* gene that includes all 4 regions conserved among human and mouse genes was divided into 2.5 kbp and 3.1 kbp segment for easy cloning. Briefly, porcine genomic DNA was extracted using a genomic DNA extraction kit (Qiagen, Germany) according to the manufacturer's protocol. The 3.1 kbp segment was cloned and inserted into a pEGFP-C2 vector (Clontech, Japan) to replace the original CMV promoter, as reported previously, to construct the pOg2 vector [21]. Next, the 2.5 kbp segment was amplified by PCR using a 2.5Up primer set (Table 1) under an initial denaturation of 3 min at 94°C, 35 cycles of 30 s at 94°C, 30 s at 54°C, 3 min at 72°C, and a final extension of 5 min at 72°C. Next, the 2.5 kbp amplicon was inserted upstream of the 3.1 kbp segment in the pOg2 vector at the* AseI* site (appears in lower-case letters in the primer in Table 1) to construct the pOG2 vector. The pOm2 vector was constructed by replacing the EGFP gene between the* Age1* and* Kpn1* site in the pOG2 vector with the corresponding mCherry CDS in the pmCherry-C1 vector (Clontech, Japan).

### 2.3. Construction of the Cell-Type-Specific Reporter Vector

A 351 bp segment of the DE1 region (primer set DE1), a 197 bp segment of the DE2 region (primer set DE2), a 164 bp segment of the PE1 region (primer set PE1), and a 759 bp segment of the PE2 region (primer set PE2) were amplified by a high fidelity npfu DNA polymerase (Enzynomics, Korea). The PCR was performed as follows: 1 cycle of 3 min at 94°C for denaturation, 35 cycles of 30 s at 94°C, 30 s at 55°C, 30 s at 72°C, and 1 final cycle of 2 min at 72°C. Then, the four amplified products were purified with a Gel Purification Kit (Bioneer, Korea) followed by digestion with the restriction enzyme* SalI* (appears in lower-case letters in each primer in Table 1). Next, a 1 : 1 mixture of DE1/DE2 was used as a template for amplifying a 554 bp segment of ΔDE with DE1-F and DE2-R primers. In the same manner, a 1 : 1 mixture of PE1/PE2 was used as the template for amplifying 888 bp of ΔPE with PE1-F and PE2-R primers. The PCR reactions were performed as follows: 1 cycle of 3 min at 94°C for denaturation, 35 cycles of 30 s at 94°C, 30 s at 65°C, 30 s at 72°C, and 1 final cycle of 2 min at 72°C.

Subsequently, the region between the* AflII* and* XmnI* sites in pOG2 was replaced by the cloned ΔDE to construct the ΔDE-pOG2 vector. The region between the* NheI* and* SmaI* sites in pOm2 was replaced by the cloned ΔPE to construct the ΔPE-pOm2 vector. The sequence between the* AgeI* and* KpnI* sites in pOm2 was replaced by the EGFP CDS region in pEGFP-C2 to construct the ΔPE-pOG2 vector. Then, an* AscI* site was added to the ΔDE-pOG2 and ΔPE-pOm2 vectors by inserting an AscI adaptor (Table 1) before the* AseI* site. Finally, the region from the AscI site to the* RsrII* site in the ΔDE-pOG2 vector and the region from the* RsrII* site to the* MluI* site in the ΔPE-pOm2 vector were ligated to complete the construction of the ΔDE-pOG2-ΔPE-pOm2 dual-fluorescence reporter vector (Figure 3(a)).

### 2.4. Cell Culture and Transfection

The F9 mouse teratocarcinoma cells were cultured in Dulbecco's modified Eagle's medium (DMEM) (Welgene Inc., Korea) supplemented with 10% fetal bovine serum (Gibco, USA), 100 units/mL of penicillin, and 100 *μ*g/mL of streptomycin (Gibco, USA). The P19 mouse carcinoma cells were cultured in alpha minimum essential medium (Gibco, USA) supplemented with 10% FBS, 1x nonessential amino acids (NEAA) (Gibco, USA), 100 units/mL of penicillin, and 100 *μ*g/mL of streptomycin. J1 mouse ESCs were cultured in DMEM supplemented with 15% ES FBS (Gibco, USA), 1x GlutaMAX (Gibco, USA), 100 *μ*M *β*-mercaptoethanol (Gibco, USA), 1 mM sodium pyruvate (Gibco, USA), 1x NEAA, 1000 units/mL of leukemia inhibitory factor (LIF) (Chemicon, USA), and 100 units/mL of penicillin and 100 *μ*g/mL of streptomycin. The cells were fed every day and split every second day with a 1 : 5 split ratio. All of the three cell lines were cultured on 0.1% (w/v) gelatin coated dishes or plates.

For transfection, briefly, 4 *μ*g of plasmid DNA was transfected into P19 cells in a 6-well plate using lipofectamine LTX (Invitrogen, USA) according to the manufacturer's protocol. Similarly, 1 *μ*g of plasmid DNA was transfected into J1 cells and F9 cells by effectene reagent (Qiagen, Germany) according to a previously described procedure [35]. For the promoter activity assay, the fluorescence intensity of transfected cells in each well was measured by VictorX multilabel readers (PerkinElmer, USA). For transgenic cell line establishment, the fluorescence-positive cell colonies were picked using a plain capillary tube (Kimble Chase, USA) under an inverted fluorescence microscope (Axiovert 200M, Carl Zeiss, Germany), cultured, and routinely passaged every 2 days.

### 2.5. Alkaline Phosphatase (AP) Staining

Alkaline phosphatase (AP) staining was performed according to a previously reported procedure [39] using the BCIP®/NBT Liquid Substrate System (Sigma, USA). First, the cells were washed with DPBS once, followed by fixation with 10% neutral buffered formalin (Sigma, USA) at room temperature for 30 min. After washing three times with Tris buffer solution, 1 mL of BCIP/NBT was added into each well of the 6-well plate. Next, the plate was slowly rocked at room temperature for approximately 30 min and then imaged.

### 2.6. Total RNA Extraction and RT-PCR

Total RNA was isolated from the transgenic cell lines using the Trizol reagent (Invitrogen, USA) according to the manufacturer's protocol. The cDNA was synthesized using TOPscript Reverse Transcription kit (Enzynomics, Korea). The expression levels of* EGFP*,* mCherry*,* Oct4*,* Sox2*,* Klf4*,* c-Myc*, and* Nanog* mRNA were detected by RT-PCR. The reaction was performed as follows: 30 cycles of 30 s for denaturation at 94°C, 30 s annealing at 62°C, and 30 s extension at 72°C. The relative expression of* GAPDH* was used as an internal control.

### 2.7. Immunofluorescent Cytochemical Staining (ICC)

The transgenic cells were seeded in a 6-well plate and cultured until cell colonies formed. Then, the cells were fixed with 4% paraformaldehyde (Sigma, USA) for 15 min at room temperature and permeabilized with 0.25% TRITON™ X-100 (Promega, USA) in DPBS for 15 min. Subsequently, the cells were blocked with 1x blocking solution (DaeMyung Science, Korea) for 1 h at room temperature and incubated with anti-Nanog (1 : 500 dilution; Abcam, UK) or anti-Oct4 (1 : 50 dilution; Abcam, UK) at 4°C in 1x blocking solution overnight. After washing three times with 1x phosphate-buffered saline with Tween 20 (LPS solution, Korea), the cells were incubated with AlexaFluor® 555-conjugated secondary antibodies (Invitrogen, USA) for 2 h at room temperature. Finally, the nuclei were stained with 5 *μ*g/mL Hoechst 33342 solution (Sigma, USA) for 5 min at room temperature. Cell images were acquired using an Axiovert 200M system (Carl Zeiss, Germany).

### 2.8. Statistical Analysis

All the experiments were performed in duplicate. The results are shown as the mean ± SEM. A *p* value < 0.05 denotes a difference possessing statistical significance. The fluorescence intensity of the pictures was converted to corrected total cell fluorescence (CTCF) by ImageJ 2x as previously described [40]. CTCF = integrated  density − (area  of  selected  cell × mean  fluorescence  of  background  readings). The data were analyzed by SPSS17.0 using Duncan's multiple comparison tests or Student's *t*-test.

## 3. Results

### 3.1. Four Conserved Regions Were Identified in the Pig* Oct4* URS

**Figure 1:**
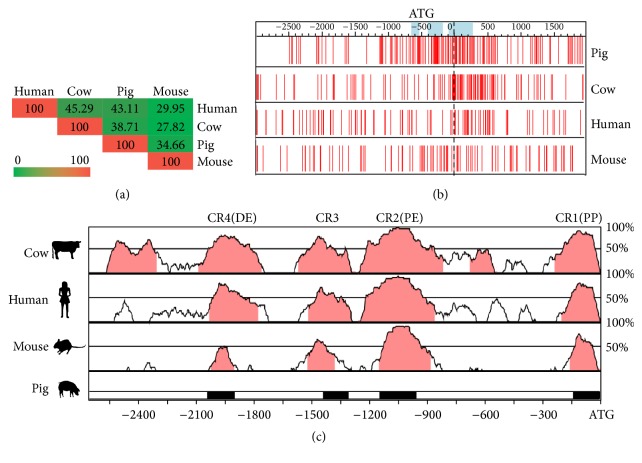
Alignment of the human, cow, mouse, and pig Oct4 gene and its upstream regulatory sequences (URSs). (a) Pig URS shares relatively low homology with the human (43.11%), cow (38.71%), and mouse (34.66%) gene, respectively. (b) The distribution of CpGs is obviously higher in the porcine Oct4 URS, and three CpG islands appear to exist only in pig but are rarely present in the human, mouse, and cow gene. Red bars at the bottom show the CpG dinucleotide. Light blue shaded regions are the predicted CpG islands. (c) Four highly conserved blocks (CR1, CR2, CR3, and CR4) in pig were found with homologies from 87.19% to 94.88% through pairwise alignments. The right axis indicates the percentage identity within a 100 bp window for each pairwise comparison, ranging from 10% to 100%. Regions sharing greater than 25% identity are shaded and the black horizontal line indicates 50% identity. CR: conserved region. DE: distal enhancer. PE: proximal enhancer. PP: minimal proximal promoter.

The URS of the porcine* Oct4* gene was successfully cloned from the umbilical cord. DNA sequence analysis showed that it shared relatively low homology with that of the human (43.11%), cow (38.71%), and mouse (34.66%) gene (Figure 1(a)). In particular, the distribution pattern of CpGs appeared unique in the porcine* Oct4* URS, and three CpG islands were only predicted around pig Oct4 proximal promoter (PP) (Figure 1(b)). However, comparative analysis identified four highly conserved regions (CR1, CR2, CR3, and CR4) and three functional elements, the distal enhancer (DE), the proximal enhancer (PE), and the PP in the pig* Oct4* URS, which were similar to those found in the human, mouse, and cow gene (Figure 1(c); see Supplementary Figure  1 in Supplementary Material available online at http://dx.doi.org/10.1155/2016/1390284). The sequence similarity in CR1, CR2, CR3, and CR4 is as high as 91.73%, 94.88%, 87.19%, and 82.86%, respectively. The CR1 region could be the most crucial part for* Oct4* expression because the minimal proximal promoter, transcriptional binding sites of Sp1/Sp3, and the hormone responsive elements (HRE) were located within it (Supplementary Figure  1). The DE was located within the CR4 region and the PE was located within the CR2 region (Supplementary Figure  1).

### 3.2. Pig* Oct4* Expression Is Regulated by Two Cell-Type-Specific Enhancers

**Figure 2:**
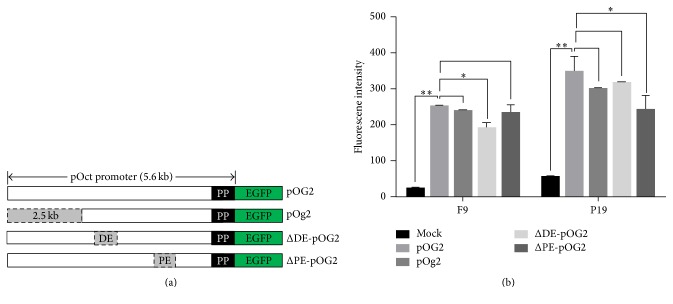
Promoter activity assay for the pig Oct4 promoter. (a) Four EGFP reporter vectors carrying different lengths of the pig Oct4 URS were constructed from a pEGFP-C2 vector. DE: distal enhancer; PE: proximal enhancer. PP: Pou5f1 proximal promoter. The deleted regions are shown in gray. (b) The removal of the 2.5 kb 5′-flanking region (pOg2 vector) had no significant impact (*p* > 0.05) on the promoter activity in each cell line. However, the promoter activity was significantly decreased in the F9 cell line (*p* < 0.05) when the DE region was removed. An obvious drop of promoter activity was detected in the P19 cell line (*p* < 0.05) when the PE region was removed. Relative activity of each promoter construct is reflected by fluorescence intensity. Data are represented as the mean ± SEM. *∗* indicates *p* value < 0.05 versus normal. *∗∗* indicates *p* value < 0.01 versus normal. Similar results were observed in more than two independent experiments.

In order to find the functional regulatory elements in the porcine* Oct4* URS, we constructed four EGFP reporter vectors carrying different* Oct4* URS lengths. Here, we used 3 types of mouse PSCs to test this reporter system. After transfection, we found that EGFP, which was controlled by the entire 5.6 kb long URS (pOG2 vector) containing all of the 4 conserved regions, was strongly expressed in F9 cells and P19 cells (Figures 2(a) and 2(b)). When the 2.5 kb 5′-flanking region was removed (pOg2 vector), there was no substantial impact on its regulatory activity. However, deletion of the DE region (ΔDE-pOG2 vector) resulted in an obvious attenuation of the fluorescence signal in F9 cells (*p* < 0.05) but had no significant effect on P19 cells (Figure 2(b)). Conversely, the fluorescence signal was significantly reduced only in P19 cells (*p* < 0.05) when transfected by the ΔPE-pOG2 vector (Figure 2(b)). These changes in EGFP expression demonstrated that the expression of* Oct4* is predominantly dependent on the DE in F9 cells rather than the PE, while PE is the predominant regulatory region in P19 cells for* Oct4* expression.

### 3.3. Establishment of Transgenic Cell Lines Stably Expressing EGFP and RFP

**Figure 3:**
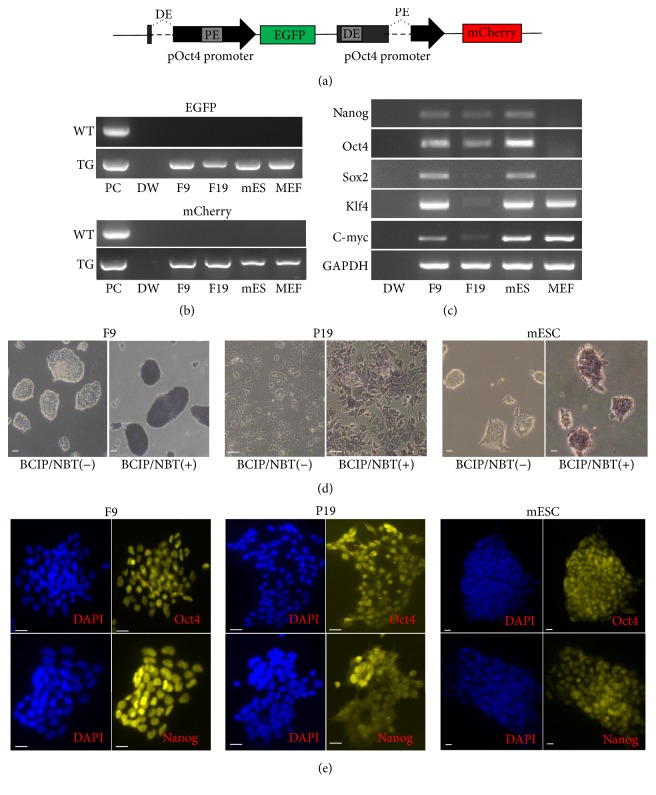
Establishment of transgenic cell lines stably expressing EGFP and mCherry (RFP). (a) A schematic illustration of the constructed ΔDE-pOG2-ΔPE-pOm2 vector using the porcine Oct4 promoter. DE: distal enhancer; PE: proximal enhancer. (b) Genotyping the Oct4 promoter-driven transgenes from genomic DNA. PC: positive control; DW: distilled water, negative control; WT: wild type; TG: transgenic cell lines. Lanes 3–6, fluorescence-positive cell lines established from wild type F9 cells, P19 cells, mESCs, and MEFs. (c) Reverse transcriptional PCR analysis of pluripotency markers in fluorescence-positive cell lines. (d) Surface alkaline phosphatase activity in transgenic F9 cells, P19 cells, and mESCs was measured using BCIP/NBT. (e) Typical pluripotency markers, Nanog and Oct4, were assayed by immunofluorescent cytochemical staining in transgenic F9 cells, P19 cells, and mESCs. Scale bars indicate 20 *μ*m.

F9 cells, P19 cells, and mESCs were transfected by the linearized dual-fluorescence reporter vector, ΔDE-pOG2-ΔPE-pOm2 (Figure 3(a)). The fluorescence-positive cells were propagated, genotyped, and established as cell lines (Figure 3(b)). To confirm the pluripotency of these transgenic cell lines, the expression of pluripotency markers (Figure 3(c)), and alkaline phosphatase activity were observed (Figure 3(d)). As expected, all of the three transgenic cell lines were shaded by the alkaline phosphatase reaction substrates, BCIP/NBT (Figure 3(e)). Immunofluorescent cytochemical staining analysis showed that the three transgenic cell lines expressed typical pluripotency markers, such as Nanog and Oct4 (Figure 3(e)).

### 3.4. Testing the Dual-Fluorescence Reporter System in Two Kinds of Defined PSCs

**Figure 4:**
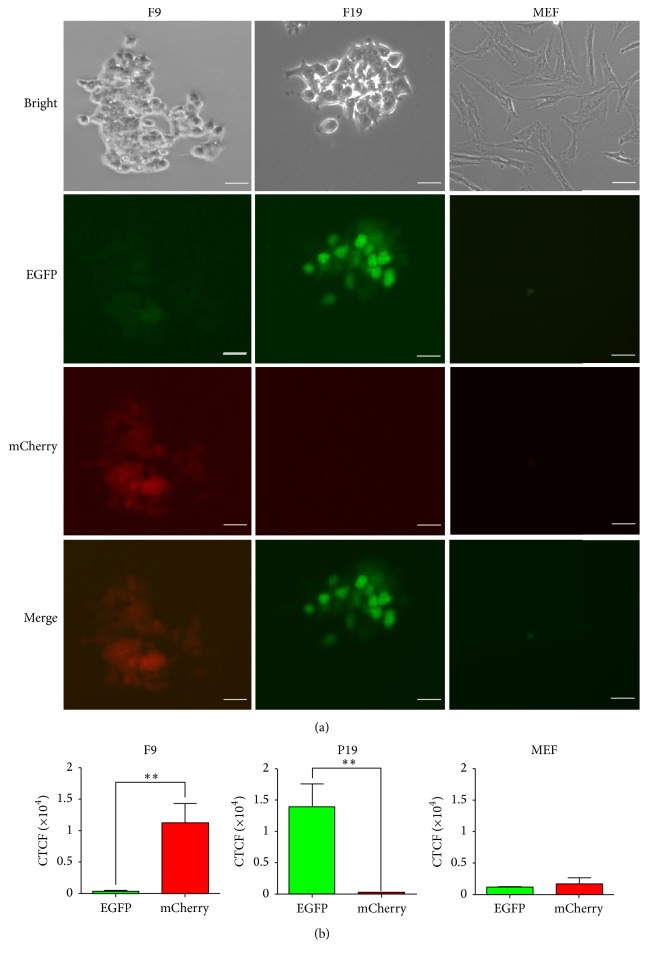
F9 cells and P19 cells exhibit completely different fluorescence signals. (a) The transgenic ESC-like F9 cells show a higher red fluorescence, while green fluorescence was primarily detected in EpiSC-like P19 cells. The negative control cell line, MEFs, exhibited neither green nor red fluorescence expression. (b) Quantitative analysis of the fluorescence intensity in a same cell clone. The fluorescence intensity of the pictures was converted to corrected total cell fluorescence (CTCF) by ImageJ 2x as previously described [35]. CTCF = integrated density  −  (area of selected cell × mean fluorescence of background readings). Data are represented as the mean ± SEM. *∗* indicates *p* value < 0.05 versus normal. *∗∗* indicates *p* value < 0.05 versus normal. Similar results were observed in more than two independent experiments. Scale bars indicate 20 *µ*m.

To determine whether the ESC-stage-like F9 cells and epiblast-stage-like P19 cells can be distinguished by the two transgenes, the fluorescence expression pattern in the 2 transgenic cell lines was determined. As we expected, the fluorescence intensity in the two types of cells was very different (Figures 4(a) and 4(b)). There was a relatively higher red fluorescence signal observed in F9 cells, while green fluorescence was primarily detected in P19 cells (Figures 4(a) and 4(b)). However, the negative control cell line, MEFs, exhibited neither green nor red fluorescence expression. This result demonstrated that our dual-fluorescence reporter could be an efficient tool to monitor the different states of PSCs.

### 3.5. Classifying mESCs from a Heterogenous Population by Dual-Fluorescence Signals

**Figure 5:**
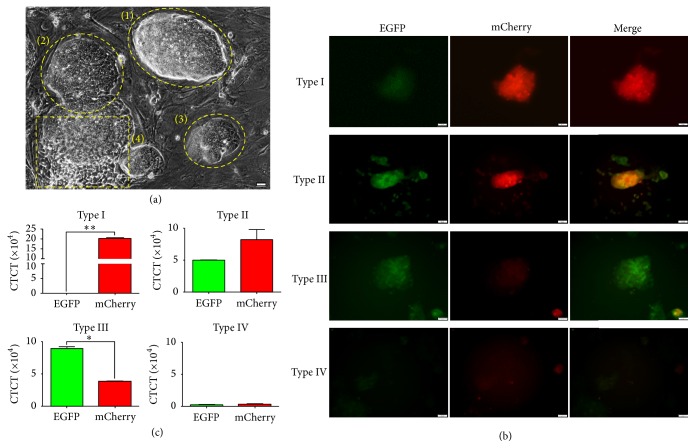
Classifying mESCs from a heterogenous population by dual-fluorescence signals. (a) When cultured* in vitro*, most ESC colonies exhibit a three-dimensional dome-like morphology (1), but a fraction of ESCs may spontaneously differentiate into flattened (2, 3) or rosette-like structures (4). (b) Four cell types were identified in transgenic mESCs. A relatively higher level of red fluorescence was detected in Type I cells. A similar level of green and red fluorescence was detected in Type II cells. A relatively higher level of green fluorescence was detected in Type III cells. Neither green nor red fluorescence is detected in Type IV cells. CTCF: corrected total cell fluorescence. (c) Quantitative analysis of the fluorescence intensity in the same cell clone. The fluorescence signal was scanned by ImageJ and converted into numeric data for comparison of cell types. Scale bars indicate 50 *µ*m. Data are represented as the mean ± SEM. *∗* indicates *p* value < 0.05 versus normal. *∗∗* indicates *p* value < 0.05 versus normal. Similar results were observed in more than two independent experiments.

Typically, mESCs are considered to be in a heterogenous state containing several intermediate unstable cell types because of spontaneous differentiation. We checked the morphology of each cell colony and found that most of them had a three-dimensional dome-like morphology with well-defined edges (Figure 5(a)(1)), but some of them were flattened colonies with smooth edges (Figure 5(a)(2) and Figure 5(a)(3)), and a small fraction of colonies appeared damaged with jagged rough edges (Figure 5(a)(4)). Accordingly, the fluorescence expression pattern in each cell colony was determined. As a result, at least four types of colonies were identified in transgenic mESCs (Figure 5(a)(2) and Figure 5(a)(3)). A relatively higher level of red fluorescence was detected in Type I colonies. Similar levels of green and red fluorescence were detected in Type II colonies. A relatively higher level of green fluorescence was detected in Type III colonies and neither green nor red fluorescence was detected in Type IV colonies. This result demonstrated that the culture population in a conventional mESC medium is heterogenous and the different subcell types can be classified by our dual-fluorescence reporter.

## 4. Discussion

The URS of a specific gene usually consists of enhancers in addition to a minimal proximal promoter (PP). An enhancer was first demonstrated in the early 1980s in SV40 [41]. It is a type of cis-regulatory element located in genomic DNA that can regulate gene expression through histone modifications [42]. Enhancers contain multiple cognate binding sites for a variety of transcription factors [43, 44]. Unlike promoters, enhancers can act near the core promoter (proximal enhancer) or over very long distances of more than several kilobases [45] or megabases [46] on the chromosome from their target gene (distal enhancer).

Some genes are only expressed in a certain cell type or tissue because their promoter activities are regulated by cell-type-specific enhancers [47, 48]. It has been reported that a powerful distal enhancer located in the URS of the* FGF4* gene can activate* FGF4* transcription in F9 cells and ESCs through two cooperative transcription factors,* Sox2* and* Oct4* [49]. A similar study of mouse* Oct4* showed that a distal enhancer could drive* Oct4* expression in undifferentiated ESCs, morula, primordial germ cells, and ICM, whereas a proximal enhancer (PE) can block its expression in the epiblast stage [33]. In humans, the* Oct4* gene was reported to be expressed in both ICM and trophectoderm [50], which is similar to rabbit [51], goat [15], and cattle, but the conclusions conflict [14]. This phenomenon can be explained as hESCs are in a metastable epiblast-like state [52], which means a given culture condition may induce them into divergent fates [53]. For example, when hESCs were epigenetically reprogrammed to a more “naïve” undifferentiated mouse-ESC-like state, they showed a regulatory pattern similar to mESCs that predominantly utilize the* Oct4* distal enhancer [54].

The expression pattern of pig* Oct4* in early embryonic development was reported to be similar to humans but different from mouse [55]. In this study, we checked the URS of the pig* Oct4* gene and found that although it is obviously different from other species, especially in the distribution of CpG dinucleotides, which are potential methylation sites that play important roles in regulating gene expression [56], the three functional regulatory boxes (DE, PE, and PP) are highly conserved. Therefore, we adopted the strategy of classifying the naïve and primed PSCs using the DE and the PE characteristics in the mouse* Oct4* URS and constructed a DE/PE-dependent dual-fluorescence reporter with the pig* Oct4* URS.

There have been many studies on pig iPSCs to date. However, some of these reported iPSC lines resemble mouse EpiSCs with a compact and flat colony morphology and dependence on bFGF [57–59], which may be in a metastable pluripotent state similar to hESCs [60]. And the others are similar to mouse ESCs that have a packed dome-shaped appearance and are primarily dependent on LIF [24, 61, 62], which are thought to be in a more “naïve” undifferentiated state [60]. More importantly, few of these putative iPSCs have been used to perform chimera studies [63]. Given this, we lack precise “authentic” or “naïve” pig PSCs for studying this reporter; instead, we first tested it in three types of defined mouse PSCs with different levels of pluripotency. As we expected, the primed-PSC-like P19 cells can be well distinguished from naïve-PSC-like F9 cells, and the heterogenous state mESCs can also be classified into at least 4 subcell types (Figures 4 and 5). Now, on one hand, we performed cell sorting through different fluorescence expression patterns and performed further analysis to verify that these 4 types of cells were different metastable state stem cells that have different pluripotency characteristics. On the other hand, we are currently testing this reporter by SCNT in pig embryo to complete the relative data for pigs.

In summary, we have cloned and characterized the pig* Oct4* URS using an approach similar to mouse. And these findings are successfully used to construct a dual-fluorescence reporter system, which has been proved effective in distinguishing three types of mouse PSCs. We expect that this reporter will also be a practical tool to distinguish porcine naïve and primed PSCs.

## Supplementary Material

Supplementary Figure 1. Alignment of the porcine Oct4 5'upstream regulatory sequences (URSs) with its human, cow and mouse orthologs showed four conserved regions(CR1, CR2, CR3, CR4), which are shaded in yellow. The sequence similarity in CR1, CR2, CR3 and CR4 are as high as 91.73%, 94.88%, 87.19%, 82.86%, respectively. The putative hormone responsive elements (HRE) regions and Sp1/Sp3 binding site (5'-GGGGGCGGGG-3') show 100% homology in all four species. A putative 2A box, a 1A box and a 1B box that similar with mouse Oct4 5' URSs have been underlined.

## Figures and Tables

**Table 1:** List of primers used in this study.

Primers	Sequences^*∗*^	Primer length (bp)	Amplicon size (bp)
2.5Up-F	5′-attaatTGTGAGCACAGTTCCATCCTGACC -3′	30	2526
2.5Up-R	5′-attaatCCAGCTGAAATGACTCCTGGGGAA -3′	30	
DE1-F	5′-GCTTGTCCTTAAGGTTCTGGGTCA-3′	24	351
DE1-R	5′-gtcgacATCTACTGCTGAGCTCCTTGGCTC-3′	30	
DE2-F	5′-gtcgacGAAGCACATCTTTCCACCCCCACC-3′	30	683
DE2-R	5′-CTCCTCTGAATCTCTTCCAGTGCC-3′	24	
PE1-F	5′-TTTTCGCTAGCCCCCCAAACAAAG-3′	24	164
PE1-R	5′-gtcgacTCACACAGAATCCCCTTCAGAGCA-3′	30	
PE2-F	5′-gtcgacTCTCCCCCCCACCTCCCTCCTT-3′	28	759
PE2-R	5′-GGTGTCTCGAGGGCGAAAGTCGGA-3′	24	
AscI adaptor	5′-TAATggcgcgccAT-3′	14	—

^*∗*^Restriction enzyme sites are shown in lowercase. F: forward; R: reverse; DE: distal enhancer; PE: proximal enhancer.
