# Supplementary material for: Construction of a Dual-Fluorescence Reporter System to Monitor the Dynamic Progression of Pluripotent Cell Differentiation
Source: Stem Cells Int. 2016 Nov 24;2016:1390284. doi: 10.1155/2016/1390284 (PMC5143739; doi:10.1155/2016/1390284)
Supplement: Supplementary file 1 — Supplementary Figure 1. Alignment of the porcine Oct4 5'upstream regulatory sequences (URSs) with its human, cow and mouse orthologs showed four conserved regions(CR1, CR2, CR3, CR4), which are shaded in yellow. The sequence similarity in CR1, CR2, CR3 and CR4 are as high as 91.73%, 94.88%, 87.19%, 82.86%, respectively. The putative hormone responsive elements (HRE) regions and Sp1/Sp3 binding site (5'-GGGGGCGGGG-3') show 100% homology in all four species. A putative 2A box, a 1A box and a 1B box that similar with mouse Oct4 5' URSs have been underlined. [file 1390284.f1.pdf]

```
Cow   ----------GGATCC-TGGGATCGCA-T-GGGATCGCAGAGTCGGACACAACTGAAGCA [  60]
Human GCTCTGTGAAGGGTCT-TGACATTGCACT-GTAATAATAAAGGTGTGTG----TGAAGTA [  60]
Pig   GTGGGGTGAGGAGTGTATGTGGAGAGTCT-GCAAACCCAGGCCTAAATTGGTTTGGGGGA [  60]
Mouse ACTCTGTAGACCAGGC-TGGCCTGGAACTCAGAAATCCACCTGCCTCTGCCTCCCAAGTG [  60]

Cow   ACTGAGCATGCACCATGCACGCACAAGACCCAAGAGAGTTGGAGGAGCCAGGTCTGATGA [ 120]
Human TCTTTTTATG----GTGACTTTCTAAAACCCAGGGAA--TCATGGGACCAGTTCTGATGA [ 120]
Pig   CTTGAAGTTTTTTAGTGACTCCCT---ACCCAAAAGAG-TGGAGAAGCCAGGTCTGATGA [ 120]
Mouse CTGGGATTAAAGCCATGAGTTGGGA--CCGCACCCGGCCCAAAGTGACTCTTAAAGGGGG [ 120]

Cow   CTCAAT-TGGCTTGCAGTCTCCTCTGG-GCCT--------------GCA----------- [ 180]
Human CTCAGCCTGGCTTCCAGTCTCCTCCAG-GCCCAACGGTGGCCCCCAGCACTGGTTGGGGC [ 180]
Pig   CTTAACCCCACTTGCAGTCTGCTCTGG-GCCT--------------GCA----------- [ 180]
Mouse CAGAGTGGCACATATTTTCAATCCTAGCACTCAGGAAGCAAAGGCTGGTGGATCTCTGTG [ 180]

Cow   ----GAGAGCTGGTCTGGGCAGAAGTGAAGCTACCTACCCTTC--AGGTTTAACAGAAGG [ 240]
Human CTGGGAGAGCTGGCCTTGGCTGAAGTGAAGCCACCTACCCTTC--AGGCATAACAGGACA [ 240]
Pig   ----GAGACCTGGCCTCTGCAGAAGTGAAGCTGCCTACACTTC--AGGCCTAACAGGAGG [ 240]
Mouse AGTTCAAGGCCAGCCTGGTCTACAGAGTGAGTTCCAAGCCATCTAAGGCTATGTAGGGAA [ 240]

Cow   GTGGGGATGGGAG---GGGAGGGTGCCTCA--GCCTTGCCAGCCTCAGCACCCCAAGACA [ 300]
Human GTGAGAA-GGAAG---GAAAGCCTGCCTCA--ACCT-------CCCATCAGCCCTGAGCA [ 300]
Pig   GTGGGGAGGAGAG---GGGAATAGGC-TCA--GCCCTGCCATGCCAAGCACCCCCAGG-- [ 300]
Mouse CCCTTGAATCAAACCCAAAAGTTAGTCTGATGATTTTCTAAGACCCAGGAGGCAAGAAAC [ 300]

Cow   TAGCTGGAAGGAACTGCAGACAAACTTAACTTTCCACTAAGGTT-CTGGGTCGG--ACTC [ 360]
Human CCCCAGAAGGGGGCCG-------------------GCTAGGAGT-CTAGGCA------TG [ 360]
Pig   ---CTGACTAGAACTCCAGACAAATTTAGCTTGTCCTTAAGGTT-CTGGGTCAG--ACCC [ 360]
Mouse TGGATCAGATGAGCCAACAGGTCTGCTGTCCCATCTCCAGGGCCACCAGGCTCACAGCTC [ 360]

Cow   CAGGCAAGCACAGGACTGATCTGGCTCAGATGTCTGGCTGACAGCCAACCAGGAACC-CT [ 420]
Human CAGG-AGGCTGACCCCTGACTGGGCTCA--TATCCAGCCACAAGGCAGCCAGGGATC-CA [ 420]
Pig   CAGGCAAGCACAGAACTGATCTGGCTCAGATGTCTGGCTACAAGCTATCCAGGAACC-CA [ 420]
Mouse GGGACCAGGCTAGGGCACATCTGTTTCA---AGCTAGTTCTAAGAAGACTTGGGACTTCA [ 420]

Cow   GGCACCCTCCCCTC--CCATC----------TCTCCCTCTGGTATGGGA----CTCTTGC [ 480]
Human GGCACCCACCCCTTGTCTGCG----------TCCCTCTCGGGAATGGGC----CTCTTGC [ 480]
Pig   GGCATCCAGCCCTCCCCAGCCCTCCCCAGGCTTTCCCTCTGGAATAGGAAGGACACTTGC [ 480]
Mouse GACAAAGTTGCTGTTAAGGACTGTATTATACTCTAGGCACGCTTAGGGCTAACCTGGTTG [ 480]

Cow   CAAG-----AAACATACCGCTTAGAGCAATCAGATTACTG-ATTGTAATCTCAAAAGGGT [ 540]
Human CCAGGCCAGAAATACACCACCTACAGTA--CAAATTAT---AATCTAAAAACAAGAGGGT [ 540]
Pig   TTAAACCAGAAACATACCATCTAGAGCAG-CTATTTATGGTGATCTAAAAAACACAGGGT [ 540]
Mouse CAAAGCCAGTCACTAGGCAGTTAAAGGACTCAGAATAT-GTCTCTTGTCCTGGCCAGTGA [ 540]

Cow   ATTGTTGAGTTGGGGG--------AAAGGCAGAAGGT--GTTTTAGGGGCTGCTGGGAAG [ 600]
Human GGTGTTGAGT-GGGGA--------AATTGGGGAAGGT--GTTTTAGGAGCCACTAGGAAA [ 600]
Pig   GCTATTTAGTCGGGGGTGGGTGG-GAAGGGAGAAGGT--GTTT-AGGGTCCGCGGGAAAG [ 600]
Mouse GTCACCAAAAGAGAAATCACAATCCATAAGACAAGGTTGGTATTGAATACAGACAGGACT [ 600]

Cow   A-----CAGCAGTG-----CAGGGGCCACATGAGAAGGGA---TTCTGAGAGCCTAGAGG [ 660]
Human AT-GGGCAGCAGGGACTCTCTGGACTGGCTTGGGAAGAGCGCTTTTGGGGAACCTGGAGG [ 660]
Pig   TCAGGGGCACAGGGGCTCTCTGGACC-ACATGGGGAGAGGGGTTTCTGGGAGGCCAGAGG [ 660]
Mouse GCTGGGCTGCAGGCA-TACTTGAACTGTGGTGGAGAGTGC---TGTCTAGGCCTTAGAGG [ 660]

Cow   GTGGCGATCGAGGCAAGGAGCATTGCTGT--AGATT---------TCCTAGGTCTGCCCC [ 720]
Human ATGGCAAGC----TGAGAAACACTGGTGTGGAGATTCCAGCCAAATCCCAGGCCTGCCCC [ 720]
Pig   GCGAGGAGC----CAAGGAGCTCAGCAGT--AGATT---------CCCTAGGCCCGCCCC [ 720]
Mouse CTGGCCCTG----GGAGGAAC-TGGGTGTGGGGAGG---------TTGTAGCCCGACCCT [ 720]
```

```
Cow   TCCCC-CTCCTCTGGGAGGCTGTC--TTCTTGG-AAACAGCAAATAAATGCATGACAAAG [ 780]
Human TCCCC-CTCCTCTGAGAGGCCGTC--TTCTTGGCAGACAGCAGAGAGATGCATGACAAAG [ 780] C
Pig   TCCCC-CTCCTCAGGGAGGCCGTC--TTCTTGGCAGACAGCAGATAGATGCATGACAAAG [ 780]
Mouse GCCCCTCCCCCCAGGGAGGTTGAGAGTTCTGGGCAGACGGCAGA----TGCATAACAAAG [ 780] R
         2A (DE)
Cow   GGACCCTGATGGCTCTGTCTTGGGGGTTGGGACATGGCTGGGGAGGGGCC-CCTCCTGG [ 840] 4
Human GTGCCGTGATGGTTCTGTCCTGGGGATTGAG---ATGGCTGGGGAGGGGCCTCCTCCTGT [ 840]
Pig   GGGCCATGATGGCTCTGTCCTGGGGGTTGGGGAGATGGCTAGGGAGGGGCC-CCTCCTGG [ 840]
Mouse GTGC-ATGATAGCTCTGCCCTGGGGGCAGAGAAGATGGTTGGGGAGGGGTC-CCTCTCGT [ 840]

Cow   TCCAAAGCACATCTC-CCCACCCCC-ACCAGGTCCCTTAATCTATCAGGTTTTTTTGGCA [ 900]
Human TCCGAAGCATGTTCCTCCCACCCCC-ACCAGGCCCCATAATCTACCTG-CCTTTTGGGCA [ 900]
Pig   TCTGAAGCACATCTT-TCCACCCCC-ACCAGGCCCCTTAATCTATCTG-CTTTTGGGGCA [ 900]
Mouse CCTAGCCCTTCCTTAATCTGCTATTGAGGAAGCTTTGTGAACTTGGCGGCTTCCAAGTCG [ 900]

Cow   --------ATTAGTGGCTTAGAGGCAAATATAGCTCCAGTTTTGCTGC---CCTGTAGAT [ 960]
Human --------GTTAAAGGCCGAGAAGTGAACACAGCTGCAACCCCACTGC---CTTGTAGAC [ 960]
Pig   --------GTTAGTAGTTTAGAGTTGAA-ATAGCTCCAGCCCTGCTGC---CCTATAAAT [ 960]
Mouse CTGCCTTTATTTAGGTCTTCCAACTAACCTATGGCACTGTTCCACAATGAATGTATAGAA [ 960]

Cow   CTT------TCATCA-GACCTATGGGAAGTCTTGAAATGCACACATATA----------- [1020]
Human CTT------CCGGCA-GACCTGTGGCAGGTATTGAAATGCACGCATACAA---------- [1020]
Pig   CTT------TCAACA-GACCTATGGGAAGTATTGAAATGCATGCACGCAA---------- [1020]
Mouse ATTGGGAGGTGAGCATGACAGAGTGGAGGAAACGGAAGATTCATGGAGAGGGCCAGAGAG [1020]

Cow   --------TTAGTTGCCCAAAAAG------TACTCACAGATGAGCTGGAACTGGGGCTAT [1080]
Human --------TTAG--GCTCAAAAAG------TCTACACAGA----CAGGAGATGGG----- [1080]
Pig   --------TTAGTCACCCCAAACG------CACAGGCCGATGGGCACTGGAAGAGATTCA [1080]
Mouse ATGGCCCCTCAGCCACCCTGGGGGATGACTTGGACCCATGTGGTAGAAGGAGGGGACTTC [1080]

Cow   GTGATACGAATTT--AGGAGAGAGTTAAGAAAAAAAAAACAGGCTCAAAAGA-------- [1140]
Human --CACACGAAC----AGAGGCAACATAAGAGTGGGGGAAAAGTCTCAAAAGA-------- [1140]
Pig   GAGGAGAAAAAGC--AAAACAAAACAACACGGACACACAAAAACCCAACAGA-------- [1140]
Mouse CACACATGTGCTATGTGTAGCTGTGTGTAGGTACATACACACCCTTAAAATAAAACGCAA [1140]

Cow   -------TTCCTAG-----ATACGACCAAGGTGAAGACAGCAGGCCACAGTCAAGAGGAC [1200]
Human -------CTCACGG-----ATGCCACCAAGATGAAGACAGCTGGCCACG-------GGAC [1200]
Pig   -------CTCAAAG-----G-ACTCCT--GGTGGAGCTAACTGGTCACAGTCTGGAGGAT [1200]
Mouse TTTTTTTTTCAAAGTCTCAGGGTGAATTTGGTGAAGTCGATGAAGCTGAGGCAGGAGAAT [1200]

Cow   CCCCAGCACCTCAGAAG-----------GCAGATG-----------CTGAGCAAATAG-- [1260]
Human ACCCATCCCCTTAGAAG-----------GCAGATAGAGCCACTGACCCCAGCAGACAA-- [1260]
Pig   GCC-AGCCCCTCAAGAC-----------AGATGCCGAGCCACTGACCCTAGCAAACAA-- [1260]
Mouse TATCAGGAGTTCAAGGGCAGCTTGTTTTATAGAGAAAGGTTCCATCTCTACCTGATGAAG [1260]

Cow   --CCTCAGACAGGGCGGGGA--------TGTAGAGA-------TGTCTAAGTCAAC--AG [1320]
Human --GCCCAGGCAGGGCTGAGCCTGGAGCCTGCAATGAGAAGCCTTACTTAAGTCGAC--AG [1320]
Pig   --CCTCAGACCCAGCCAAGA--------TGAAGAGG-------TGTCTAGGTCCGC--AG [1320]
Mouse ACTACCATCAAGAGACACCCCCGCCCCCCAGGGCACCTAGAGCCACTGACCCTAGCCAAC [1320]

Cow   AGGTC---CATGC----TCCAGGCTCAGGATTCTGGCCTTCCAAGGTGTATGGAGCTCTG [1380]
Human AGGTCAG-CGTGCCCAGTCCAGACCTGGCCTTCTGGCCTTCGAAGCTGTGGGGAGCCCTG [1380]
Pig   AGGTC---TGTGT----CCCAGTCTCAGGAGTCTGGCCTCC-AAACTGTAGGAAGCTCTG [1380]
Mouse AGCTCAGGCGGGCTGGGCCCAGGCTCAGAACTCTGTCCTGGCTATGTACACTGTGGGGTG [1380]

Cow   GTTCACAGCCTTTCCGGAGT--CCCCCCAATTCAC-----CTCGGGGCCTTCATTTGGGG [1440]
Human GCCCAGAGCCCCCTCTGGAG--CCCCCAGACTTAC-----CCCAGGCCCTCCACTGAGAT [1440]
Pig   ATCCATGGCTTCTCTGGAGAGCCCCCCTCACTCAGGTTCACCTGGGGCCTTCGTTTAGGG [1440]
Mouse CTCTGGG---CTTTTTGAGG--CTGTGTGATTCAC-----CCTGGGGCCTTCGTTCAGAG [1440]
```

```
Cow   CAAGTTTGGGGAGCAGACAGACAAACATCATTCCTAGCAGACAGGCAATCTGAAAGCTAT [1500]
Human CAAGTTTTGGGAGCAGACAGACAAACATCATCCCTCACAGACAGGCATTCCGTTGGCTAT [1500]  C
Pig   CAAGTTGGGGGAGCAGACAGACAAACATCATCCCCAGCAGACAGCCAGTCTGAAAGCTAT [1500]
Mouse CATGGTGTAGGAGCAGACAGACAAACACCATCCCTTGCAGACAGGCACTCTGAGGGCTAT [1500]  R

Cow   TCTCTTGCAAACAGAGAATAAGCACTAAGCCTTCAGTGTGAGCC--------CTCAGGAC [1560]  3
Human TCTCTTGCAAACAGA-ATCAAGCACTAGACCAGCAGCATGAGCCTCAGGATACTCAGGCC [1560]
Pig   TCTCTTGCAAACAGA-ATCAAGCACTAGGCCAGCAGCCTGAGCC---------TCAGGAC [1560]
Mouse TCTCTTGCAAAGATA-ACTAAGCACCAGGCCAGTAATGGGATCC---------TCAGACT [1560]

Cow   CAGCCCAGA--------CCCTGTGGGAGAGCTTAGGTCAGGCTTCTTGCACCTCCACCCC [1620]
Human AGGCCCAGAAAAACAGACCCTGAAGGGGAGCTTAGGGCAGCCTTCCTGCACGCCT-CCAC [1620]
Pig   AGACCCAGAAAAATAGACCCTGTGGGAGAGCTTAGGGCAGGATTCCTGCACCCCCTCCCC [1620]
Mouse GGGCCCAGAAAA----CCACTCTAGGGAAGTTCAGGGTAGGCTCTCTGCACCCCCTCCTC [1620]

Cow   AAGTCGCTCTCCTCTTCCT-CCTCATCTTTTTGCCAGCC----CCCCTAAACAAGGC--- [1680]
Human AAATCACTCTCCACCTCCT-CTGCGTCTTTCTGCCAGCCAGCCCCACTAAACAAAGCACA [1680]
Pig   AA-TCGCAGTTCACCCCCTTCTGCATCTTTTCGCTAGCC-----CCCCAAACAAAG---- [1680]
Mouse ----CTAATCCCGTCTCCT-TAGTGTCTTTCCGCCAGCA------CAGGAATGGGG---- [1680]

Cow   ---------CTGGGGGGCGGGGGGAGGGGTAGAAGATGAGGCTGAATGCCTGCGTCCTCT [1740]
Human TCCCTCAATCTGCCAGGCTCGGGGAGGGACGCACGATGAAGCTGGACGCCTGAGTCCCCC [1740]
Pig   ---------------------------------------GCCTGGACGCCTCAGTCCTCT [1740]
Mouse -----------------------GAGGGGTGGGTGACGAGGATGAACACCGGAGTCC-CT [1740]
          1A (PE)
Cow   ACAGG-----GGGACGGGATACCTAGGCCCCAGTGGGCGGCCCT--------GTCTGAGG [1800]
Human AGAGGAAGGAGGAACTAGATACCTAGGTCCCTGTGGGGGGCCCTTGGTGCCCGTCTGAGG [1800]
Pig   AGAGGG----GGGACAGGATACCTAGGTCCCAGTGGGGGGCCCT--------GTCTGAGG [1800]
Mouse GGAGGAAGG-GAAGCAGGGTATCT-----CCA---------------------TCTGAGG [1800]

Cow   CTCACAGTCTTTGAGGGGTTGGTGGGGGGGTGGTTGCTGCAGGAGCTCTTTTAGCTGCTC [1860]
Human CTCA--GTCTTTGAGGGGATTGCAGAGGGG-GGTTGCTG--GAGCTCCTTTTAGCGTCTC [1860]
Pig   CTCA--GTCTTTGAGGGGAT---GGGGGTGTTGTTGCTG---GAGCTCTTTTAGCTGCTC [1860]
Mouse CTCT--GTCTTTGAGGAGA----GGTGGAG----AGCTG--------------------- [1860]

Cow   TGAGGGGGATTCT-TAGGAGGGGATTGGGGCT----GGGGG-TTGGGGGGCAGGAAGCTG [1920]
Human TGAAGGGGATTCTGTGTGAGGGGATTGGGACT----GGGGGGTTGGGGAGCAGGAAGCAG [1920]
Pig   TGAAGGGGATTCTGTGTGAGGGGATTGGGGCT----GGGGGGTTGGGGGGCAGGAAGCTG [1920]
Mouse ---GGGAAGTCTTGTGTGAGGGGATTGGGGCTCAGGAGGGGGTTGGGGAGCAGGAAGTTG [1920]

Cow   TCCCCAGGGGAGCCATCCAGGCCCATTCAAGGGTTGAGCACTTGTTTAGGGTTAGAGCTG [1980]  C
Human TCCCCAGGGGAGCCATCCAGGCCCATTCAAGGGTTGAGCACTTGTTTAGGGTTAGAGCTG [1980]
Pig   TCCCCAGGGGAGCCATCCAGGCCCATTCAAGGGTTGAGCACTTGTTTAGGGTTAGAGCTG [1980]  R
Mouse TCCCCAGGGGAGCCATCCTGGCCCATTCAAGGGTTGAGTACTTGTTTAGGGTTAGAGCTG [1980]
                                1B (PE)                                  2
Cow   CCCCCTCTGGGGACCAGGATTGTCCAGCCAAGGCCATTGTCCGGGCCCCTTCCCCCAGTC [2040]
Human CCCCCTCTGGGGACCGGGATTGTCCAGCCAAGGCCATTGTCCTGCCCCCTTCCCCCAGTC [2040]
Pig   CCCCCTCTGGGGACCAGGATTGTCCAGCCAAGGCCATTGTCCGGCCCCCTTCCCCCAGTC [2040]
Mouse CCCCCTCTGGGGACCAGGATTGTCCAGCCAAGGCCATTGTCCTGCCCCCTTCCCCCAGTC [2040]

Cow   CCTCCCAAGCCCCTTTGAACCTGAAGTCAGATATTT-TT-TTTCTCCCC-T-TCTACCTC [2100]
Human CCTCCCAGGCTTCTTTGAACCTGAAGTCAGATATTT-TT-TCTCCACAC-C-CCCCACCC [2100]
Pig   CCTCCCAAGCCTCTTTGAACCTGAAGTCAGATATTT-TTCTCTCCCCCC-CACCTCCCTC [2100]
Mouse CCTCCCAGGCCCCTTTGAACCTGAAGTCAGATATTTCTTCTCTCTACCCACCTCCCACCC [2100]

Cow   CTAGGCTTTTCCCACCCAGGGCCTAGGGCTGGAGGTCTGGGGT-GGTAGGAGGGGG-AGG [2160]
Human CCTGGTTTTCCCCACCCAGGGCCTAGGGCTGGAGGCCTGGGCCAGGGAGGTGGGGG-AGG [2160]
Pig   CTTGGCTTTCCCCACCCAGGGCCTAGGGGTGGAGGCCCAGATT-GGGAGGTGGGGG-AGG [2160]
Mouse GTTGGGTTTCTCCACCCAGGAACTAGG-CTGGAAGCCTGGGATGAGGAGGTGGGGGGAGG [2160]
```

```
Cow   GAGAACCT--TCAACTGTGGTTCTAGATATTTGGGTCTCTG--AAGAGG----GGGGCAA [2220]
Human GAGAACGGGGCCTACCGTGGTATTAGATGTCTGAGTTTTGGTTGAGAGG----GGAGCAA [2220]
Pig   GAGAACAG--TCAACTATGGGGCTAGATATTTGGGTCCCTGAAGGGGGGCTGGGGGACAA [2220]
Mouse GAGAACTG--AGAATCTTGAGGAAAGAGGCCCCGGCCTTAACTGTGAGG-------GGAT [2220]

Cow   GGAACTTGATGCACG---GGACCCACAGTGAGGGACCCGTGGTGTGGTGT--GTCCAATC [2280]
Human GGAACCTGATGTGCA---GGTTCCATAGTGGAGGGGGCCCAAAGCGG-GT--GTCTTATC [2280]
Pig   GGAACCTGATGTGCGCGGGGACCCACAGCGGGGGACCTGCGAGCGGGTGTCCGATTGATT [2280]
Mouse GGAGCCTGG-GTGCA---GGTCTTATGGGGGTTGGGGGGTGGT-TAGTGTCTAATCTACC [2280]

Cow   CCTCTGGCTGGACAAAGGTAGGGAGACTCAGGCCTAGTCAGTCCAGAGCCTGGCCCC--- [2340]
Human ACTCTGTTTCAGCAAAGGTTGGGAAACTGAGGCCCAGTCAGTCCAAAGTCTGGTCCCTTG [2340]
Pig   CCTCTGCCTGCACAAAGATTGGGAGACTCAGGCCCAGTCCATC--GAGCTTGATCCCTGG [2340]
Mouse AACCTGGAC-AACACAAGATGGAATACT-GTGCTCTGAAAACGCAGAGCCAGCACTTCT- [2340]

Cow   ----GAAAGTAGGGTTTCCATCCCTGGGTCTGGTAGGTGAGGGGCCTCTGGAGTCAG--- [2400]
Human AAGGGGAAGTAGGGAC-CAACCCCTTAGTCTGTTAGATGAGGAGAGTCTGGAGTCTGATT [2400]
Pig   AAGGGAAAATGGGGGTTCCATCCCTGGGTCTGGTGGA-AGGGAGGCCCCGGAACCCG--- [2400]
Mouse --------CTGGGGTCTCTGGGGACATATCTGGTTG-------GGGCTCGGGGTCCC--- [2400]

Cow   ----------GAAAGCTGGGG--------------------ATGGAATGGAAGC-TGGTC [2460]
Human CTGGAAGACGGAGGGGTGGGGGGATGGGGGGGTGGGGGGATATAGCACGGAGGCCTTGTC [2460]
Pig   ----------GAAAACTG----------------------TACGGAATGGAAGC-CCGTG [2460]
Mouse -----------------------------------------ATGGTGTAGAGCC----TC [2460]

Cow   TGGCAGCCTGCCCCTGGTGATGGGGTGGATTTTAGCAGGCTGGGCAGAGGGGTACCAGGC [2520]
Human TGGCAGTCTACTCTTGAAGATGGGGTGAAATTTGGCAGGCTGGGCAGATGG-TGCCAGGC [2520]
Pig   TGGCAGTCTGCCCCTGGTGA-GGGGTGGAATCTAATAGGCTGGGCGGATGGTTGCTGGGC [2520]
Mouse TAA------ACTCTGGAGGACTGGAGGTGCAATGGCTGTCTTGTC---CTGGCCTTGGAC [2520]

Cow   ATCACAGTTTTGAAAAG--TGAAAGTGTTAGTCA-T----GTCCGACTCTTTTTGACCC- [2580]
Human ACCCAGGCTGCGGGGTGGCTGGATTTGGCCAGTA-TCGGGATGGGAATGCCTAGGATTCT [2580]
Pig   ATCGCAGCTTTGGGGTGCCGGAATCTGGCCAGTAATCTAGTTGGGAATGCCTAGGTTCC- [2580]
Mouse AT--GGGCT--GAAATA-CTGGGTTCACCCA----------------TATCTAGGACTC- [2580]

Cow   --ACAGATTGTAGCCCGCC----GAGTTCCTCTGTCCATGGGATTTCCCAG-GCAAGCAT [2640]
Human GGATGGATCGGGGGAAGGCATAAGGGAGCAGCTGGCCATTGTGCTTATGGCTGTTGATGC [2640]
Pig   ---CGGACTGGGGGT--------------------------------------------- [2640]
Mouse ---TAGACGGGTGGG--------------------------------------------- [2640]

Cow   ACTGGAGTGGGTCGCCATTTCTTTCTTCAGGAGATCTTC----------------CCAAC [2700]
Human ATTGAGGGATAGCGCCACACACACATTCAATAAATTTGAGGAGCTGAGAGGGTGACTGGC [2700]
Pig   ------------------------------------------------------------ [2700]
Mouse ------------------------------------------------------------ [2700]

Cow   CC----AGGGATACAACCAG-GGTCTCTGGCATTGCAGGCAGATTCTTTATCATCTGAGC [2760]
Human CCCTGAAGGCACAGTGCCAGAGGTCTGTGGAGAGGGGGTCAAGCACCTGGGTTCCTGAAG [2760]
Pig   ------------------------------------------------------------ [2760]
Mouse ------------------------------------------------------------ [2760]

Cow   CACCAGG-------GAAACGAATCTGCCCAGTTG------------TCTAAA-------- [2820]
Human AACATGGAGGTGTGGGAGTGATTCCAGACAGCTGGGATGTGCAGAGCCTGAGAGAGTGCC [2820]
Pig   ---------------------------------------------------GAG-------- [2820]
Mouse ------------------------------------------------------------ [2820]

Cow   -----------TGGGAACTGC---CTAGGT------------TCCTGGACTGGGATGCT [2880]
Human AGGGAGCGGGTTGGGAGTTGAAAGTTGGGTGTGGTGGCTCACGCCTTTAATCATGACACT [2880]
Pig   ------------------------------------------------------------ [2880]
Mouse ------------------------------------------------------------ [2880]
```

```
Cow   GGAGGACAACGGGGAAGGAGCCTGTTAAGAATTGAGGGGTAGCCCCAGTGGGACTTGACA [2940]
Human GGGCGGCAGAGGCGGGAGGATTTCTTGAGGACAGGAATTCAAGACCAGCCTGGGT-AACA [2940]
Pig   ----GGCAGAGAG------------CAGGAATTGAGGAGTAGCTCCGGCAGGACTTAGCA [2940]
Mouse ---------------------TAAGCAAGAACTGAGGAGTGGCCCCAGAAATAATTGGCA [2940]

Cow   CATACAGCATAAATGTGTTTGAGGAGTTGAGAGGGTGGCTGGTCTCAGGTGCCAGGGTGT [3000]
Human TAGCAAGGCCCCATCTCTACTAAAAATAAAAAAACTAACAGGGCACAGTGGTCCAAGCCT [3000]
Pig   CAGACA--CCAGACCTGTGTGAGGACCTGAGAGGGTCGCTGGG----GTCCCTTGAGGAG [3000]
Mouse CA-------CGAACATTCAATGGATGTTTTAGGCTCTCCAGAG----GATGGCTGAGTGG [3000]

Cow   CTGGAGAGGGGTCCTGAAGAACGTGCAG--GGGGTATGGAAGGGACCCCTCACCCCTGGT [3060]
Human GTAGTCCCAGCCACTTAGGAGGCTGGAGCAGAAGGATTGCTTTGGCCCAGTAGATCGAGG [3060]
Pig   ACAGTGCCAGGGTCTTCGAAGAGGGGTCC-AACACCTGGCTC---CCCGACAGCCCCAAT [3060]
Mouse GCTGTAAGGACAGGCCGAGAGGGTGCAGT-GCCAACAGGCTTTG--TGGTGCGATGGGGC [3060]

Cow   GTATGT-GGGCCTGG-GGAGGGCTGGGCAGC----CTGTTGGGAGCTGGAAGTGAAGGCC [3120]
Human CTACATTGAGCCATC-ATTGTACTCCACTGCACTCCAGTCTGGGCAACAAAGTGAGACCC [3120]
Pig   GTGCACAGAGCAGTGGAGAGGGCCGGGCGGC----CGGTTGGGAGTTGGAGGTGAAGGCC [3120]
Mouse ATCCGAGCAACTGGTTTGTGAGGTGTCCGGT----GACCCAAGGCAGGGGTGAGAGGACC [3120]

Cow   CGCATGGGGGACCTGCACCGAGAG-------TCTAGGAGTCTGGGGCCTGGA-----GAG [3180]
Human TGTCTTAAAAAATAAAAATAAAAAAAGTTTCTGTGGGGGACCTGCACTGAGGTCCTGGAG [3180]
Pig   -GCATGGGGGACCTGCACCAAGGG-------CCTGGGGACC--GCA-----------GAG [3180]
Mouse ----TTGAAGGTTGAAAATGAAGG-----CCTCCTGGGGTCCCGTCCTAAG------GGT [3180]

Cow   GGGCCTGGGTGGAGATCCCTGGCTTTCCCCTTCCA--GACACCACCGCCACCAGCA---G [3240]
Human GGGCGCCAGTTGTGTCTCCCGGTTTTCCCCTTCCACAGACACCATTGCCACCACCATTAG [3240]
Pig   GCGCCCGGGCGGACCTCTCCGACTTTCGCCCTCGA--GACACCACCGCCACCAGCC---A [3240]
Mouse TGTCCTGTCCAGACGTCCCCAACCTCCGTCTGGAA--GACACA----------------G [3240]

Cow   GCAAACACCCTCCGCCTCAGTTTCTCCCACCCCCACCGTCCCTTCCCCCCACCCATCCAG [3300]
Human GCAAACATCCTTCGCCTCAGTTTCTCC---CCCCACC-TCCCTCTCCTCCACCCATCCAG [3300]
Pig   GCAAACACCCTCCGCCTCAGTTTCTCCCACCCCCACCGACCC-CTCCCCCACCCATCCAG [3300]
Mouse GCAGATAGCGCTCGCCTCAGTTTCTCCCACCCCCACAGCTCTGCTCCTCCACCCACCCAG [3300]

Cow   GGGGCGGGGCCAGAGGTCAAGGCTAGTGGGTGGGATTGGGGAGGGAGAGAGGTGTTGAGC [3360]   C
Human GGGGCGGGGCCAGAGGTCAAGGCTAGTGGGTGGGACTGGGGAGGGAGAGAGGGGTTGAGT [3360]
Pig   GGGGCGGGGCCAGAGGTCAAGGCTAGTGGGTGGGATTGGGGAGGGAGAGAGGTGTCGAGC [3360]   R
Mouse GGGGCGGGGCCAGAGGTCAAGGCTAGAGGGTGGGATTGGGGAGGGAGAG----GTGAAAC [3360]
       Sp1/Sp3      HRE                                                   1
Cow   AGTCTC-TAGGAGATCCCTCGTTTTCCTAGGCCCCCGGCTCGGGGTGCCTTCCTTCCCC [3419]
Human AGTCCC-TTCGCAAGCCCTCATTTCACCAGGCCCCCGGCTTGGGGCGCCTTCCTTCCCC [3419]
Pig   AGTCCCCTTGGAGAGCCCTGGTTTTACTGGGCCCCCGGCTTGGGGCGCCTTCCTTCCCC [3419]
Mouse CGTCCC-TAGGTGAGCCGTCTTTCCACCAGGCCCCCGGCTCGGGGTGCCCACCTTCCCC [3419]
```

Supplementary Figure 1. Alignment of porcine Oct4 5' upstream regulatory sequences (URSs) with its human, cow and mouse orthologs. Four conserved region appear in shades of yellow. The HRE site, 2A box in DE, 1A and 1B box in PE have been underlined. The regulatory protein binding sites are circled.
